# The Effect of EX‐B8 Acupressure on Labor Pain: A Randomized, Single‐Blind, Sham‐Controlled Trial

**DOI:** 10.1155/prm/7873155

**Published:** 2025-02-24

**Authors:** Hannaneh Azadeh, Reza Heshmat, Malihe Nasiri, Fatemeh Azarkish, Sedigheh Sedigh Mobarakabadi

**Affiliations:** ^1^ Department of Midwifery and Reproductive Health, School of Nursing and Midwifery, Student Research Committee, Shahid Beheshti University of Medical Sciences, Tehran, Iran, sbmu.ac.ir; ^2^ Acupuncture, International School of Lyon, Lyon, France; ^3^ Department of Biostatistics, School of Nursing and Midwifery, Shahid Beheshti University of Medical Sciences, Tehran, Iran, sbmu.ac.ir; ^4^ Department of Midwifery, Tropical and Communicable Diseases Research Center, Iranshahr University of Medical Sciences, Iranshahr, Sistan and Baluchistan, Iran, irshums.ac.ir; ^5^ Department of Midwifery and Reproductive Health, Midwifery and Reproductive Health Research Center, School of Nursing and Midwifery, Shahid Beheshti University of Medical Sciences, Tehran, Iran, sbmu.ac.ir

**Keywords:** acupressure, EX-B8 point, labor pain, primiparous women, sham point

## Abstract

**Context:**

Intense, uncontrolled pain during labor can have negative outcomes for both the mother and the baby, but this can be prevented by utilizing pain‐relieving techniques. Childbirth is a natural physiological process, and it is important to prioritize non‐pharmacological methods such as acupressure in managing the associated pain.

**Objective:**

The present research aims to determine the effects of acupressure on the eighth point of the extra‐back meridian (EX‐B8) for pain relief during childbirth in primiparous women.

**Design:**

This study was a randomized, single‐blind, sham‐controlled trial.

**Setting:**

This study was conducted at Shahid Rasulullah Hospital in Nikshahr, Sistan and Baluchistan Province, Iran.

**Patients or Other Participants:**

Ninety primiparous mothers in the active phase of the first stage of labor were selected and randomly divided into three groups: acupressure on EX‐B8 (*n* = 30), sham (*n* = 30), and control group (*n* = 30).

**Intervention(s):**

The acupressure and sham groups received acupressure for 20 min during their uterine contractions at three different time points: when cervical dilatation was at 4‐5 cm, 6‐7 cm, and 8–10 cm, totaling 60 min. The control group received routine labor care.

**Main Outcome Measure(s):**

Pain intensity was assessed using a Numerical Rating Scale (NRS) before, 10 min after, and 20 min after the start of the intervention at three different time points.

**Results:**

Pain intensity was significantly lower in the EX‐B8 acupressure group compared to the sham and control groups at all three time points of the intervention (*p* < 0.05). In the EX‐B8 group, the greatest amount of pain relief was achieved during dilatation of 8–10 cm, compared to dilatations of 4‐5 and 6‐7 cm (*p* = 0.0001). Maternal and neonatal outcomes did not differ significantly between the three groups (*p* > 0.05).

**Conclusion:**

The current study found that applying acupressure on EX‐B8 effectively reduced pain during labor. Acupressure on this point can be recommended as an effective, low‐cost, and accessible pain‐relieving technique, especially at the end of the active phase of the first stage of labor. Further studies are needed to determine why acupressure on this point is more effective at the end of the active phase of labor.

**Trial Registration:** Iranian Registry of Clinical Trials: IRCT20211108053006N1

## 1. Introduction

Labor pain is one of the strongest types of pain that most women experience during their lifetime [1]. The surge in catecholamine secretion caused by labor pain [2] can lead to poor maternal and neonatal outcomes [3, 4], which can be prevented with pain relief [5]. Currently, both pharmacological and non‐pharmacological methods are used to reduce pain during labor. In recent years, the general public has perceived childbirth as a natural physiological process and using non‐pharmacological methods to control labor pain has become preferable [2]. Acupressure was ranked third in the evaluation of the acceptability and effectiveness of ten non‐pharmacological methods of pain relief during labor [6].

Acupressure is rooted in traditional Chinese medicine (TCM) and is similar to acupuncture in its ability to strengthen calm, promote health, and treat illnesses. According to TCM, the body has energy meridians through which vital energy (qi) flows. The twelve main meridians connect the organs or special networks of the organs, forming a system of communication throughout the body. Acupuncture points are located along these meridians and can be stimulated to alleviate pain or disease and balance the flow of vital energy [7].

There are 12 main meridians in the body, but new acupoints have been discovered and named “Extra points.” Most of these Extra points are not located on any of the 12 meridians and have been grouped according to their position. These include the EX‐HN extra point (head and neck), EX‐CA extra point (chest and stomach), EX‐B extra point (back), EX‐UE extra point (upper extremity), and EX‐LE extra point (lower extremity). The EX‐B extra point includes 28 points that are situated at 0.5 cun intervals on a horizontal line from the sides to the lower lumbar spinous process, between C1 and S4 [7].

The Chinese term for EX‐B8 is Shiqizhui and it is located in the depression below the spinous process of the 5th lumbar vertebra. Recent studies suggest that acupressure on this acupoint has therapeutic effects on gynecologic diseases, such as irregular menstrual cycles, PMS, primary dysmenorrhea (PD), and metrorrhagia [8]. Ma et al. and Li et al. found that acupressure on the EX‐B8 acupoint provides effective analgesia for dysmenorrhea. The noteworthy point is that an immediate pain relief is experienced by stimulating this point [9, 10].

Our knowledge of the effects of the extra meridian points during labor is limited. Increased pharmacological and invasive interventions during vaginal birth require further research to enhance labor and improve the experience of labor pain for mothers. Therefore, this study aims to investigate the effects of acupressure on the EX‐B8 point for pain relief and labor outcomes in primiparous mothers.

## 2. Methods

### 2.1. Research Setting and Participants

This was a randomized, single‐blind, sham‐controlled trial (RCT). Primiparous mothers who visited Shahid Rasulullah Hospital in Nikshahr, Sistan and Baluchistan Province, Iran, between April 5 and September 1, 2022, were included in the study. The inclusion criteria consisted of individuals aged 19–35 years, primiparous, with a gestational age of 37–41 weeks, singleton pregnancy, vertex position, 4‐5 cm dilated, experiencing at least 3 contractions every 10 min, having an appropriate pelvic situation, not using analgesic drugs (chemical or herbal) in the last 8 h, and without chronic diseases, known mental disorders, pregnancy complications, or obstetric complications at the time of study enrollment. The exclusion criteria included the mother’s unwillingness to continue participation in the study.

### 2.2. Pilot Study

Initially, the researcher in charge of carrying out the intervention (first author) learned acupressure and how to determine acupoints from an acupuncturist (second author). In the next stage, an experimental study was conducted to determine the accuracy of the acupressure method. Eligible participants who were willing to take part in the study were selected and divided into two groups: one receiving acupressure on the EX‐B8 point (inferior L5, midline) and the other receiving sham acupressure (inferior L5, 3 cun to the right or left horizontally). The participants were fully briefed on the study objectives, methods, the Numerical Rating Scale (NRS) for pain and uterine contractions, and how to report the pain score to the researcher before and after the intervention. Acupressure was performed on five mothers at dilatations of “4‐5,” “6‐7,” and 8–10 cm on the EX‐B8 pressure point. This was done during their uterine contractions, and their pain intensity was recorded before and after the intervention. In five other mothers, acupressure was performed on sham points in the same manner. The intervention was successfully performed in all positions, as long as the researcher accurately identified the intended acupoint. As a result, obstacles, issues, and the feasibility of implementing the intervention were investigated.

### 2.3. Sample Size and Randomization

Primiparous mothers who arrived at the maternity ward to give birth to their baby and were willing to participate in the study were selected as the sample, as long as they met the inclusion criteria. The minimum sample size was set at 27 per group, determined using the ANOVA table with *α* = 0.05, *β* = 0.10, and Δ/*σ* = 1. To accommodate for potential dropouts, the sample size was increased to 30 per group. Sampling began purposively, with participants then randomly assigned to groups using the random number function in Excel. Prior to the intervention, an Excel output prepared by third author, containing a random sequence of participants numbered from 1 to 90, was placed inside an envelope. The researcher responsible for conducting the intervention (first author) remained unaware of the group allocation of the research subjects until after they were assigned to their respective groups.

### 2.4. The Intervention

Uterine contractions were monitored before the intervention began. The researcher was informed of the start and stop times during the acupressure intervention. Participants were briefed on the NRS and provided with a sample for better understanding. In the EX‐B8 group, participants were asked to assume their desired positions. The researcher then positioned herself behind the parturient. When the parturient indicated the start of her contractions, the researcher applied pressure with her right thumb on the parturient’s EX‐B8 point. The pressure was applied deeply and in a circular manner around the EX‐B8 point until a change in color occurred in the researcher’s nail.

The intervention lasted for 20 min from the beginning of the contraction. Pressure application ended after the contraction ended and resumed at the start of the next contraction. Acupressure was applied at EX‐B8 point during cervical dilatations of 4‐5 cm, 6‐7 cm, and 8–10 cm for 20 min each, totaling 60 min. Acupressure was administered to the sham group near the EX‐B8 acupoint, 3 cun to the right or left horizontally, on a point without therapeutic effects, following the same procedure as in the EX‐B8 group. In the control group, the researcher provided emotional support at the mother’s bedside from the time of 4‐centimeter dilatation of the cervix until the end of birth, but did not apply pressure on the EX‐B8 or sham points.

### 2.5. Outcome Measurements

The intensity of pain (as the primary outcome) was measured on the NRS ranging from 0 (no pain) to 10 (worst pain imaginable). Pain intensity was assessed at three time points during cervical dilatation: 4‐5 cm, 6‐7 cm, and 8–10 cm, both before and 10 min and 20 min after the intervention in the acupressure groups. Pain intensity was also evaluated in the control group at the same intervals as the acupressure groups using the NRS. Secondary outcomes, such as the type of birth (vaginal, instrumental delivery, and C‐section) and 1‐ and 5‐minute Apgar scores, were also recorded.

### 2.6. Data Analysis

Data were analyzed using SPSS Version 22 software. The Friedman test was conducted to compare intragroup changes in pain, while the Wilcoxon test was used for pairwise intragroup comparisons. Since the data distribution was not normal, the Kruskal–Wallis test was performed to compare intergroup pain relief. The Mann–Whitney test was conducted for pairwise comparisons between groups.

## 3. Results

### 3.1. Demographics

**Figure 1:**
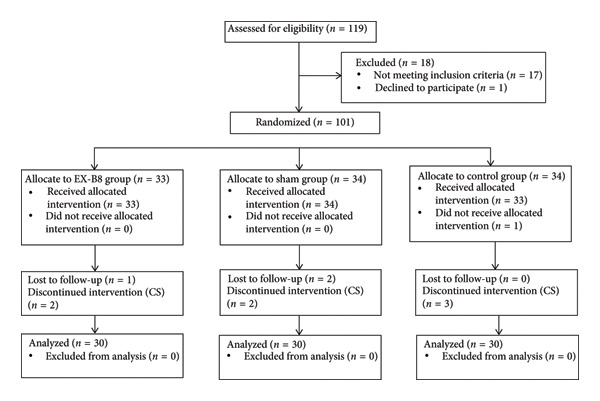
Participants’ flow diagram.

Overall, 90 participants remained in the study until the end (Figure 1). All participants were married and lived with their spouses. Table 1 shows no significant differences among the three groups in terms of demographic variables. No significant differences were observed between the three groups in terms of receiving prenatal care (*p* = 0.53), planned pregnancy (*p* = 1.000), and the baby’s gender being desirable (*p* = 0.75). The position of mothers during the interventions did not differ in the first, second, and third intervention in the three groups (*p* = 0.88, *p* = 0.60, *p* = 0.07, respectively). Most of the mothers in all groups chose to lean forward while sitting (56%), with the researcher sitting behind them. Table 2 presents the baseline details of the mothers participating in the study.

**Table 1:** Demographic characteristics.

Characteristics/groups	EX‐B8 (*n* = 30)	Sham (*n* = 30)	Control (*n* = 30)	*p*
Age (years) (mean ± SD)	25.27 ± 4.98	26.90 ± 4.97	25.00 ± 4.16	0.28^a^
Women’s education (%)				
Elementary and secondary	73.33	80.00	60.00	0.448^b^
High school and university	26.66	20.00	40.00	
Women’s occupation (%)				
Housewife	83.30	83.30	73.30	0.53^b^
Employed	16.70	16.70	26.70	
Housing (%)				
Rental	86.60	83.33	83.33	0.91^b^
Private	13.40	16.66	16.66	
Insurance (%)				
Yes	86.70	93.30	86.70	0.63^b^
No	13.30	6.70	13.30	
Income satisfaction (%)				
Less than enough	16.66	6.66	10.00	0.45^b^
Enough	83.33	93.33	90.00	
Ethnicity (%)				
Baluch	70.00	83.30	60.00	0.36^b^
Persian	16.70	10.00	26.70	
Other	13.30	6.70	13.30	

^a^Kruskal–Wallis test.

^b^Chi‐square test.

**Table 2:** Baseline characteristics.

Characteristics/groups	EX‐B8 (*n* = 30)	Sham (*n* = 30)	Control (*n* = 30)	*p*
Gestational age (weeks) (mean ± SD)	39.33 ± 1.29	38.56 ± 1.43	38.76 ± 1.36	0.10^a^
Pattern of uterus contractions				
Frequency (mean ± SD)	3.63 ± 0.96	3.20 ± 1.29	3.40 ± 1.24	0.37^a^
Duration (second) (mean ± SD)	48.17 ± 7.25	48.17 ± 8.03	50.50 ± 8.02	0.47^a^
Amniotic membrane (%)				
Intact	30.00	30.00	36.70	0.40^b^
Ruptured	70.00	70.00	63.30	
Use of 10 unit oxytocin (minute) (mean ± SD) (%)	84.33 ± 21.53 (50.00)	95.00 ± 20.20 (56.70)	95.77 ± 24.48 (56.70)	0.33^a^

^a^Kruskal–Wallis test.

^b^Chi‐square test.

### 3.2. Pain Outcomes

Table 3 shows the mean pain intensity in all the three groups. A statistically significant difference was observed between three groups regarding pain relief. The Mann–Whitney test revealed a significant difference between the EX‐B8 and control groups (*p* = 0.04, *p* < 0.0001, *p* < 0.0001), the sham and control groups (*p* = 0.88, *p* = 0.24, *p* = 0.01), and the EX‐B8 and sham control groups (*p* = 0.009, *p* = 0.001, *p* < 0.001) for pain relief at the three time points of the intervention, respectively. The Friedman test also revealed a significant intragroup difference in the EX‐B8 group throughout the three time points of the intervention. The Wilcoxon test demonstrated a significant difference after the intervention in the first and third stages (*p* = 0.0001) and the second and third stages (*p* = 0.007) in the EX‐B8 group. The Wilcoxon test also revealed a significant difference in pain relief before and 20 min after the start of the intervention, as well as 10 and 20 min after the start of the intervention in the EX‐B8 group at 4‐5 cm of dilatation (*p* = 0.05, *p* = 0.05). There was also a significant difference in reduced pain intensity before and 10 min after the start of the intervention, before and 20 min after the start of the intervention, and 10 and 20 min after the start of the intervention in the EX‐B8 group, respectively, at 6‐7‐ and 8–10‐centimeter dilatation (*p* = 0.01, *p* < 0.01, *p* < 0.01, *p* < 0.01, *p* < 0.01, *p* = 0.07).

**Table 3:** Comparison of pain score before and after each intervention and pain relief among the groups in three dilations.

Stages	4‐5 cm				*p* ^a^	6‐7 cm				*p* ^a^	8–10 cm				*p* ^a^
Groups	Before	After 10 min	After 20 min	Change		Before	After 10 min	After 20 min	Change		Before	After 10 min	After 20 min	Change	
EX‐B8 (*n* = 30)	7.43 ± 1.00	7.43 ± 0.97	6.97 ± 0.96	−0.46 ± 0.81	0.003	8.17 ± 0.74	7.77 ± 0.97	7.23 ± 1.00	−0.93 ± 063	0.001	8.50 ± 0.77	7.97 ± 0.80	7.03 ± 0.76	−1.46 ± 0.89	0.001
Sham (*n* = 30)	7.43 ± 1.04	7.70 ± 0.79	7.60 ± 0.85	0.16 ± 0.79	0.20	8.23 ± 0.72	8.33 ± 0.84	8.20 ± 0.99	−0.03 ± 0.92	0.54	8.77 ± 0.62	8.90 ± 0.88	8.63 ± 0.96	0.33 ± 0.71	0.14
Control (*n* = 30)	7.07 ± 1.08	7.90 ± 1.02	7.47 ± 1.10	0.40 ± 0.77	0.001	7.93 ± 0.82	8.57 ± 0.93	8.40 ± 1.03	0.46 ± 0.68	0.001	8.77 ± 0.72	9.43 ± 0.56	9.10 ± 0.84	−0.13 ± 0.81	0.001
*p* ^b^	0.52	0.14	0.02	0.001		0.54	0.004	0.001	0.03		0.27	0.001	0.001	0.001	

^a^Friedman’s test.

^b^Kruskal–Wallis test.

### 3.3. Other Findings

Table 4 displays the secondary outcomes compared among the three groups. In the EX‐B8 group, two participants had C‐sections, as did two in the sham group, and three in the control group. No statistically significant differences were observed between the groups (*p* = 0.94). One newborn in the EX‐B8 group and one in the control group required resuscitation. No significant differences were observed in the 1‐ and 5‐minute Apgar scores among the three groups (*p* = 0.80). There were no statistically significant differences in pethidine administration among the groups (*p* = 0.55).

**Table 4:** Comparison of secondary outcome variables and satisfaction among the groups.

Outcomes/groups	EX‐B8 (*n* = 33)	Sham (*n* = 34)	Control (*n* = 34)	*p*
Type of birth (%)				
Vaginal	90.00	86.70	83.30	0.94^a^
Cesarean section	6.70	6.70	10.00	
Operative delivery	3.30	6.70	6.70	
Birth weight (gram) (mean ± SD)	2985.00 ± 321.67	2878.33 ± 300.19	3051.67 ± 330.74	0.10^b^
Apgar score at first min (mean ± SD)	8.73 ± 0.52	8.73 ± 0.52	8.63 ± 0.66	0.80^b^
Apgar score at fifth min (mean ± SD)	9.73 ± 0.52	9.73 ± 0.52	9.67 ± 0.54	0.80^b^

^a^Chi‐square test.

^b^Kruskal–Wallis test.

## 4. Discussion

This single‐blind randomized controlled trial (RCT) investigated the impact of acupressure on EX‐B8 for pain relief during the first stage of labor and compared the outcomes with those of sham and control groups. A significant reduction in pain intensity was observed after each stage of the intervention in the EX‐B8 group compared to the other two groups. The EX‐B8 point was found to be effective in alleviating pain during the first stage of labor, particularly at 8–10 cm of dilatation. Further signs of pain relief were also observed after 10 min from the start of acupressure, and greater pain relief was observed with the continuation of the intervention. No adverse effects on maternal and fetal outcomes were observed with acupressure on the EX‐B8 point.

Our study found that acupressure at the EX‐B8 point at three different time points during labor (4‐5 cm, 6‐7 cm, and 8–10 cm dilatation) reduced labor pain. Bu and Ma et al. reported that acupuncture at EX‐B8 was effective in relieving PD [9, 11]. In dysmenorrhea, myometrial contractions and vasoconstriction result in uterine ischemia and the production of anaerobic metabolites. These metabolites cause hypersensitivity of pain fibers, ultimately leading to the sensation of pain. Pain in dysmenorrhea is typically felt in the lower abdomen, pelvis, lower back, and inner thighs [12]. Similarly, during the active phase of the first stage of labor, women typically experience pain in the lower abdomen, iliac crests, and back, which may extend to the thighs and perineum by the end of this stage [13]. Considering the similarities in pain mechanisms and location between PD and childbirth, these studies’ findings support our results. Stux and Pomeranz propose that stimulating acupoints near the pain center can result in soothing effects through three centers: the spinal cord, midbrain, and hypothalamic‐pituitary axis, ultimately leading to effective analgesic effects [7].

In the present study, the average reduction in pain intensity at 4‐5 cm, 6‐7 cm, and 8–10 cm of dilatation was −0.46 ± 0.81, −0.93 ± 0.63, and −1.46 ± 0.89, respectively. After conducting pairwise comparisons of the groups, we identified that the most significant reduction in pain occurred at 8–10 cm of dilatation. Ozgoli et al. compared the effects of stimulating the LI4 and BL32 acupoints on alleviating labor pain. Similar to our study, they reported the mean changes in pain intensity as measured on the NRS at 4‐5 cm, 6‐7 cm, and 8–10 cm dilatations to be −3.06 ± 1.25, −3.46 ± 1.37, and −3.31 ± 1.69 in the LI4 group and −3.69 ± 1.37, −4.34 ± 1.60, and −3.83 ± 1.75 in the BL32 group, respectively. After pairwise comparison of the groups, it was found that the greatest pain reduction had occurred in the BL32 group at 6‐7 cm of dilatation [14]. Additionally, when comparing the level of pain reduction in our study with the study by Ozgoli et al., we observed that the mean pain reduction was lower in our study.

The study conducted by Torkyan et al. focused on the impact of GB21 acupoints on pain relief during the first stage of labor. The results indicated that the mean changes in pain intensity, as measured on the NRS at 4‐5 cm, 6‐7 cm, and 8–10 cm of dilatations, were −1.42 ± 0.91, −1.17 ± 0.92, and −1.11 ± 0.53, respectively [15]. The mean pain reduction observed at 8–10 cm of dilatation was consistent with the findings of our study. Ma et al. found that performing EX‐B8 point acupuncture within 5 min after the onset of pain resulted in more effective pain relief than acupuncture before the onset of dysmenorrhea [9]. A comparison of the mean pain reduction between our study and others suggests that the effectiveness of different acupoints in alleviating pain may vary. It is conceivable that distinct stages of dilatation necessitate the stimulation of specific acupoints.

Another important point to note is that oxytocin was administered to 3.4% of the participants in the GB21 group in the study conducted by Torkyan et al. to aid in labor progression [15]. In our current study, 50% of the participants in the EX‐B8 group received oxytocin. However, there were no statistically significant differences in this aspect among the three groups. Conell‐Price et al. developed and validated a dynamic model to explain labor progression based on pain. Their study revealed that 48% of women who received oxytocin reported higher levels of pain at the beginning of labor but did not experience accelerated or increased pain levels compared to those who did not receive oxytocin therapy [16]. In a meta‐analysis conducted by Wei et al., it was found that women who received oxytocin during the first stage of labor reported higher levels of pain and discomfort [17]. Therefore, the lower level of pain reduction observed in our study compared to the other two studies may be attributed to the higher levels of oxytocin administered.

In our study, we observed a significant reduction in pain levels 10 min after the start of the intervention in the EX‐B8 group during the second and third time points of intervention, compared to the sham and control groups. Ozgoli, Sedigh, and Heshmat conducted a study on the effects of acupressure on the right‐hand Hugo point on the intensity of labor pain. They found that significant analgesia was achieved following the stimulation of the Hugo point during 10 min, compared to the control group. The researchers noted that continued acupressure resulted in greater pain relief [18]. These findings are consistent with our results, as our data analysis showed a significant reduction in pain after 10 min at 6‐7 cm and 8–10 cm of dilatations, with further pain reduction observed after 20 min. Further research is needed to determine if continued intervention after 20 min can produce a stronger analgesic effect.

The results of our study showed similar levels of pain intensity at all three dilatations before initiating the intervention. The similar pain intensity before the intervention at 6‐7 cm and 8–10 cm dilatation indicates that the analgesic effect achieved at the end of the 20‐min intervention is not sustainable. Studies by Ozgoli et al. and Torkyan et al., who also examined the effect of acupressure at three dilatations, reported results consistent with the present findings [14, 15]. It appears that ongoing acupressure is necessary to achieve pain relief as labor progresses and pain intensifies over time.

In this study, we included a sham group to explore the placebo effect of acupressure, which did not demonstrate a significant reduction in pain intensity. To address potential bias from researcher presence, a control group was also included. The control group underwent the same procedures as the acupressure groups. A notable difference in pain intensity was observed, attributed to an increase in average pain levels. Torkyan et al., who utilized acupressure on GB21 points, obtained similar results to ours concerning the control group [15]. However, Ozgoli et al. noted a modest yet significant decrease in pain intensity in the control group for dilations of 4‐5 cm and 6‐7 cm, but not for 8–10 cm [14]. Our study’s results regarding the sham and control groups suggest that the significant pain reduction in the EX‐B8 point acupressure group is not influenced by researcher presence or psychological effects of receiving a calming intervention.

The results of the current study did not reveal any statistically significant differences in terms of maternal and neonatal outcomes. Torkyan et al., Ozgoli et al., and Akbarzadeh et al. all reported no maternal complications, effects on fetal distress, or changes in the Apgar score [14, 15, 19].

The present study is one of the few dedicated to evaluating the effects of stimulating the EX‐B8 acupoint on pain intensity during labor. One of the strengths of the study was having both sham and control groups. However, one limitation was the excessive use of oxytocin to progress labor and accelerate birth in the research setting. Further studies are required to determine the effects of oxytocin administration on the pain reduction caused by acupressure. One additional limitation of this study was that we aimed to examine the impact of applying pressure on point EX‐B8 during the active phase of the first stage of labor. As part of the study, thumb pressure was applied for a total of 60 min (3 sessions of 20 min each). However, it is important to note that this duration can be challenging and taxing on the therapist’s thumb. To address this issue, pressure was only applied during contractions.

The present study shows that applying acupressure to the EX‐B8 point effectively alleviates labor pain. No adverse effects on the mother or baby were observed with this intervention. Therefore, acupressure on EX‐B8 can safely and effectively relieve labor pain. Future studies should compare the pain relief achieved by stimulating the EX‐B8 point with and without oxytocin administration. Additionally, further research is needed to understand why acupressure on this point is more effective toward the end of the active phase of labor.

**Nomenclature**
EX‐B8: Eighth point of the extra‐back meridian
RCT: Randomized controlled trial
NRS: Numerical rating scale

## Ethics Statement

The research was approved by the Ethics Committee of Shahid Beheshti University of Medical Sciences (IR.SBMU.PHARMACY.REC1400.202). All the participants were briefed on the study objectives, methods, and the confidentiality of their data. The eligible participants were also assured that they were free to leave the study at any time, and the researcher was blinded to the type of intervention and study outcomes. The participants were randomly divided into three groups, and informed consent was obtained from each of them. No changes were made to the research setting except for the addition of acupressure intervention for pain relief. All the mothers participating in the study received the necessary routine care and therapy.

## Conflicts of Interest

The authors declare no conflicts of interest.

## Author Contributions

Hannaneh Azadeh: software, data curation, validation, investigation, and draft preparation. Reza Heshmat: conceptualization, methodology, and draft preparation. Malihe Nasiri: formal analysis and writing manuscript. Fatemeh Azarkish: methodology and writing manuscript. Sedigheh Sedigh Mobarakabadi: supervision, conceptualization, methodology, and writing–reviewing and editing manuscript.

## Funding

This research did not receive any specific grant from funding agencies in the public, commercial, or not‐for‐profit sectors.

## Data Availability

The data that support the findings of this study are available on request from the corresponding author.
