# Correction to “The Effect of EX‐B8 Acupressure on Labor Pain: A Randomized, Single‐Blind, Sham‐Controlled Trial”

**DOI:** 10.1155/prm/9832031

**Published:** 2025-12-29

H. Azadeh, R. Heshmat, M. Nasiri, F. Azarkish, and S. S. Mobarakabadi, “The Effect of EX‐B8 Acupressure on Labor Pain: A Randomized, Single‐Blind, Sham‐Controlled Trial,” *Pain Research and Management* 2025 (2025): 7873155, https://doi.org/10.1155/prm/7873155.

In the article titled “The Effect of EX‐B8 Acupressure on Labor Pain: A Randomized, Single‐Blind, Sham‐Controlled Trial,” there was an error in an author’s name; Hannaned Azadeh should be Hannaneh Azadeh. The correct author list is shown below:

Hannaneh Azadeh,^1^ Reza Heshmat,^2^ Malihe Nasiri,^3^ Fatemeh Azarkish,^4^ and Sedigheh Sedigh Mobarakabadi^5^

^1^Department of Midwifery and Reproductive Health, School of Nursing and Midwifery, Student Research Committee, Shahid Beheshti University of Medical Sciences, Tehran, Iran

^2^Acupuncture, International School of Lyon, Lyon, France

^3^Department of Biostatistics, School of Nursing and Midwifery, Shahid Beheshti University of Medical Sciences, Tehran, Iran

^4^Department of Midwifery, Tropical and Communicable Diseases Research Center, Iranshahr University of Medical Sciences, Sistan and Baluchistan, Iranshahr, Iran

^5^Department of Midwifery and Reproductive Health, Midwifery and Reproductive Health Research Center, School of Nursing and Midwifery, Shahid Beheshti University of Medical Sciences, Tehran, Iran

The online version of this article has been corrected accordingly.

We apologize for these errors.

